# Supplementary material for: ntLink: A Toolkit for De Novo Genome Assembly Scaffolding and Mapping Using Long Reads
Source: Curr Protoc. 2023 Apr 11;3(4):e733. doi: 10.1002/cpz1.733 (PMC10091225; doi:10.1002/cpz1.733)
Supplement: Supplementary file 1 [file CPZ1-3-0-s001.pdf]

ntLink: a toolkit for *de novo* genome assembly scaffolding and mapping using long reads

Lauren Coombe, René L. Warren, Johnathan Wong, Vladimir Nikolic, Inanc Birol

Supplementary Tables

Supplementary Table 1. Accessions for the long-read datasets listed in Table 1.

Species	Fold coverage of long reads	Accession(s)
Caenorhabditis elegans	93	SRR10028109
Oryza sativa	62	SRR10589512- SRR10589711
Solanum lycopersicum	72	ERR6668574 (Subsampled to ~72-fold)
Homo sapiens	50	s3://ont-open-data/gm24385_2020.11/analysis/r9.4.1/20201026_1644_2-E5-H5_PAG07162_d7f262d5/guppy_v4.0.11_r9.4.1_hac_prom/basecalls.fastq.gz

Supplementary Table 2. Baseline assemblies used for the example ntLink runs listed in Table 1.

Species	Sequencing reads used for baseline assembly	Assembler used for baseline assembly	Baseline assembly parameters
Caenorhabditis elegans	Long reads (SRR10028109)	Flye (v2.9.1)	--nano-raw SRR10028109.fastq -g100m -t48
Oryza sativa	Long reads (SRR10589512-SRR10589711)	GoldRush (v1.0.0) goldtigs*	G=373e6 P=10 a=1 m=20000 polisher_mapper=minimap2 span=2 dist=500 t=48 polisher=goldrush-edit
Solanum lycopersicum	Long reads (ERR6668574, subsampled to 72-fold)	GoldRush (v1.0.0) goldtigs*	G=824e6 P=15 a=1 m=20000 polisher_mapper=minimap2 span=2 dist=500 t=48 polisher=goldrush-edit
Homo sapiens	Short reads (SRR11321732)	ABySS (v2.2.3)	j=48 k=112 kc=3 B=150G l=40 s=1000 v=-v q=15 H=4 S=1000-10000 N=9 N=5-20 pelib1_de=-n5

*GoldRush goldtigs are the polished, corrected golden path sequences generated from the GoldRush *de novo* genome assembler.
